# Virucidal activity of Formulation I of the World Health Organization's alcohol-based handrubs: impact of changes in key ingredient levels and test parameters

**DOI:** 10.1186/2047-2994-2-34

**Published:** 2013-12-12

**Authors:** Jochen Steinmann, Britta Becker, Birte Bischoff, Thomas Magulski, Joerg Steinmann, Eike Steinmann

**Affiliations:** 1 MikroLab GmbH, Norderoog 2, D-28259 Bremen, Germany; 2 Institute of Medical Microbiology, University Hospital Essen, Virchowstrasse 179, D-45147 Essen, Germany; 3 Division of Experimental Virology, TWINCORE, Centre for Experimental and Clinical Infection Research; a joint venture between the Medical School Hannover (MHH) and the Helmholtz Centre for Infection Research (HZI), Feodor-Lynen-Str. 7, D-30625 Hannover, Germany

**Keywords:** Alcohol-based handrubs, Hand antisepsis, Infection prevention and control, WHO Formulation I, Virucidal activity, Quantitative suspension test, prEN 14476:2011

## Abstract

**Background:**

A recently modified World Health Organization (WHO) Formulation I was examined as 80% and 97% solutions against poliovirus type 1, adenovirus type 5 and murine norovirus according to the new European Norm prEN 14476:2011. In a previous study the unmodified WHO Formulation I had failed to demonstrate a sufficient activity against poliovirus type 1 according to the European Norm EN 14476–2007 whereas a sufficient activity was seen with adeno- and norovirus.

**Findings:**

The modified WHO Formulation I demonstrated a virucidal activity against all 3 test viruses of the European Norm prEN 14476:2011 under clean conditions. This was achieved as 80% solution against adeno- and norovirus within 30 seconds and as 97% solution against poliovirus within 60 seconds. Testing the unmodified WHO Formulation I against poliovirus type 1 in the 97% assay of the European Norm prEN 14476:2011 an identical activity could be demonstrated. When comparing the 80% and the 97% assay of the European Norm prEN 14476:2011 the modified WHO Formulation I as 80% solution was active against adenovirus type 5 within 30 seconds whereas the 97% solution failed within 2 minutes exposure time.

**Conclusions:**

The technical possibility in the new European Norm prEN 14476:2011 allows testing a ready-to-use disinfectant as 97% solution and is responsible for the new virucidal claim of the modified WHO Formulation I. In contrast to the improvements with poliovirus type 1 the activity against adenovirus type 5 decreased when increasing the test concentration from 80% to 97%.

## Findings

In 2009, the World Health Organization (WHO) published two alcohol-based Formulations to be used in healthcare settings where commercial hand rubs are too expensive or not available [1]. Later it was shown that both Formulations were not able to fulfil the requirements of the European Norm (EN) 12971 [2] for surgical hand treatment [3]. In addition, both Formulations did not meet the requirements for virucidal activity according to EN 14476:2007–02 [4] with polio- and adenovirus [5]. Formulation I, based on 80% (vol/vol) ethanol, demonstrated a much better activity than Formulation II (based on 75% (vol/vol) isopropyl alcohol) against the test viruses. But even Formulation I failed to achieve a 4 log_10_ reduction of poliovirus titre [4].

Meanwhile, it was found that glycerol significantly decreases the efficacy of alcohol-based surgical hand rubs [6]. In order to reach an efficacy for the surgical hand treatment, the WHO Formulations were proposed to be modified with higher alcohol (mass instead of volume percentage, now approximately 85% ethanol) and lower glycerol concentration (0.725%) now fulfilling the requirements of the EN 12791 [7]. In parallel testing virucidal activity of disinfectants was changed in Europe. In the prEN 14476:2011 [8] it is now allowed to examine ready-to-use products as 97% solutions. Furthermore, murine norovirus (MNV) is included as test virus as surrogate for human norovirus. The soil load used was 0.03% bovine serum albumin (BSA) as final concentration. A 4 log_10_ reduction of virus titre is still necessary to demonstrate virucidal activity [8].

Following these recent developments the aim of the present study was to examine the modified WHO Formulation I against the test viruses mentioned in the prEN 14476:2011. Can the modification of the ingredients and/ or an increase of the test concentration cause a complete virucidal activity which was missed in our previous study [5]?

The prEN 14476:2011 [8] was chosen as a test method evaluating the activity of the old and the modified WHO Formulations I against the poliovirus type 1 strain LSc-2ab, adenovirus type 5 strain Adenoid 75 and MNV strain S99.

Poliovirus was cultivated with buffalo green monkey (BGM) cells, whereas A-549 cells (human lung carcinoma cell line) were used for titration of adenovirus. MNV test suspension and virus detection were prepared with RAW 264.7 cells (a macrophage-like, Abelson leukemia virus transformed cell line derived from BALB/c mice). All tests were conducted in accordance with the European Norm in duplicates. Eight parts by volume of the Formulation were mixed with one part by volume of the test virus suspension and one part by volume of 0.3% BSA resulting in an 80% concentration of the Formulation I. Testing the 97% solution one part by volume of the test virus suspension and the one part by volume of the interfering substance (5x) were mixed with 9.7 parts by volume of Formulation I. Activity was immediately stopped by serial dilution with virus controls after the longest exposure time. Infectivity was determined in a micro-procedure by endpoint dilution titration (TCID_50_). Titre reduction calculated as the difference between the virus titre of the water control and the Formulations is presented as reduction factor (RF).

**Figure 1:**
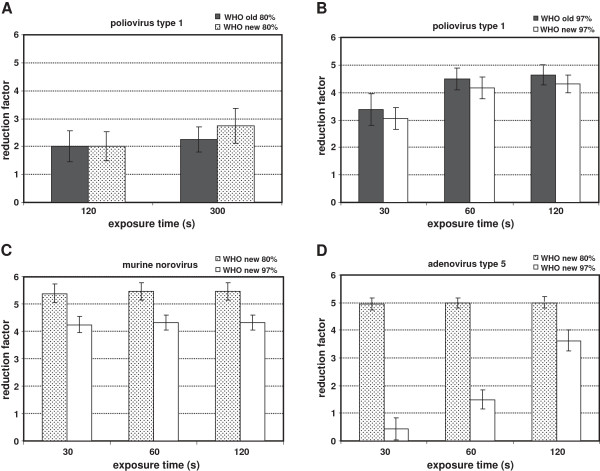
**Activity of the old (poliovirus only) and the proposed new WHO Formulation I against polio- (A, B), noro- (C) and adenovirus (D) tested as 80% and 97% solutions under clean conditions.** Virucidal activity is given as reduction factor wit 95% confidence interval.

The modified WHO Formulation I when tested as 80% solution was not active against poliovirus type 1 within 120 and 300 seconds exposure time (Figure 1A) despite the increased ethanol content. But increasing the test concentration to 97% an activity was found with the old and the new Formulations within 60 seconds (Figure 1B).

Testing the modified WHO Formulation I against MNV the 80% solution demonstrated virucidal activity (RF ≥ 5.38) within 30 seconds (Figure 1C). This was already previously shown with PBS as interfering substance (5). After this exposure time a RF of ≥ 4.25 was determined with the 97% solution (Figure 1C).

In addition, the modified WHO Formulation I was also active against adenovirus type 5 as 80% solution within 30 seconds (RF ≥ 4.94). Increasing the concentration in the test mixture to 97% no sufficient activity was found after 120 seconds as the longest exposure time in the prEN 14476:2011 (Figure 1D).

Nevertheless, in this study a sufficient activity of the modified WHO Formulation I as 80% solution against noro- and adenovirus and as 97% solution against poliovirus was shown despite the change of the interfering substance from PBS (EN 14476:2007) to clean conditions (prEN 14476:2011). Due to the reduced amount of test virus suspension (100 μl instead 1 ml) virus titres used for the calculation of the RF in the 97% assay were lower than in the 80% assay resulting consequently also in lower RFs when testing an identical test virus suspension. Despite the lowering of virus titre when examining 97% solution a 4 log_10_ reduction can be detected with the poliovirus within 60 seconds. Surprisingly, in the test with the adenovirus type 5 the change from 80% to the 97% concentration is combined with a decrease of viral activity. At the moment there is no clear explanation of this phenomenon in the quantitative suspension test. In addition, the practical consequences for hand hygiene also remain obscure when an activity against adenovirus at the human hands is required. There are no data available concerning a reduced activity of ethanol against human adenovirus at the human hands based on the established test methods like the ASTM Standards E 2011 – 09 (using the entire hands) and E 1838 – 10 (using the fingerpads) when increasing the concentration of the active ingredient [9,10]. In a previous study with feline calicivirus (FCV) as former surrogate of norovirus it was seen that in the suspension assay ethanol achieved a maximum of activity at a concentration of 67% v/v and with the fingerpad method 70% v/v ethanol was more active than 90% v/v against FCV [11].

In summary, in the prEN 14474:2011 there is the new possibility to test a ready-to-use disinfectant as 97% solution when a sufficient activity is not available as 80% [8]. For claiming an activity against poliovirus not the modified amount of ethanol and glycerol is responsible for demonstration a RF of 4 log_10_ steps but exclusively the increase of test concentration in the test mixture. Therefore, after this technical change the modified WHO Formulation I can be declared as virucidal in the quantitative suspension test strengthening the importance to use this Formulation in hospitals where virus infections often occur. Furthermore, testing with 97% is more realistic than the 80% assay comparing with practical use where handrubs are used undiluted. But the missing activity against adenovirus type 5 for the 97% concentration in the quantitative suspension assay within 2 minutes exposure time and the impact of this finding on daily practice has still to be clarified.

The quantitative suspension has still great limitations when creating a virucidal claim of a handrub and making use-recommendations. But a sufficient in vivo efficacy of a handrub cannot be expected if no activity can be found in the suspension test. Therefore, performing the prEN 14476:2011 is still necessary before starting an assay with artificially contaminated hands of volunteers. After demonstrating the activity in the suspension test the value of the modified WHO Formulation I to inactivate chosen test viruses at the artificially contaminated hand is still waiting.

## Competing interests

All authors report no conflicts of interest relevant to this article.

## Authors’ contributions

JS, ES and JS made substantial contributions to conception and design. BB, BB and TM carried out the tests. JS, ES and JS were involved in drafting the manuscript, and all authors gave final approval of the version to be published.
